# Relationship between Urinary AD7c-NTP with Cerebral Microbleeds Based on APOE Genotype

**DOI:** 10.1155/2021/3928060

**Published:** 2021-10-08

**Authors:** Yong-peng Yu, Ya-li Zheng

**Affiliations:** Department of Neurology, Weihai Central Hospital Affiliated to Qingdao University and Weifang Medical College, Weihai, Shandong 264400, China

## Abstract

**Objective:**

This study was performed to investigate the association between urinary Alzheimer-associated neuronal thread protein (AD7c-NTP) with cerebral microbleeds (CMBs) based on the apolipoprotein E (*APOE*) genotypes.

**Methods:**

A total of 471 patients with acute cerebral infarction screened by magnetic sensitive imaging were enrolled in this study. Among them, twenty-seven cases of mixed CMBs were excluded. A total of 444 patients were divided into two groups according to the presence or absence of CMBs: CMBs group (*n* = 92) and noncerebral microbleeds group (nCMBs) (*n* = 352). Urine AD7c-NTP levels were measured using a human enzyme immunoassay kit.

**Results:**

In patients with lobar CMBs, there was an interaction between urine AD7c-NTP levels and *APOE* genotypes (*p* = 0.01). In patients with *APOE ε3/ε3* allele, the odds ratio of lobar CMBs per standard deviation of urinary AD7c-NTP levels was 0.92 (95% CI: 0.70-1.19). In patients with *APOE ε2+* or *ε4+* allele, the multivariate-corrected odds ratio of lobar CMBs per standard deviation of urinary AD7c-NTP levels was 2.95 (95% CI: 1.38-6.27).

**Conclusion:**

A higher level of urinary AD7c-NTP is involved in lobar CMBs, not deep CMBs.

## 1. Introduction

Cerebral microbleeds (CMBs) are small cerebral vascular lesions characterized by microbleeds [1]. The occurrence and significance of CMBs in various cerebrovascular diseases are increasingly being paid more attention and have gradually become an important issue in the current research field of cerebrovascular diseases. At present, genetic factors are considered to be involved in the occurrence and development of cerebral vascular disease including CMBs. In terms of genetics, the apolipoprotein E (*APOE*) *ε*4 allele is by far the only genetic factor known to increase the risk of CMBs [2–4]. In 2008, an epidemiological study of CMBs in Iceland found that the *APOE ε4/ε4* genotype was associated with the occurrence of CMBs [5]. De La Monte and Wands further found that *APOE ε*4 was closely related to lobar CMBs [6]. Baseline grading of white matter hyperintensity, lacunar infarction, and *APOE ε2* carrier status can predict CMBs events [7]. Patients with cerebral amyloid angiopathy (CAA) have a higher incidence of CMBs in the lobe, and *APOE ε4* carriers are more likely to have multiple lobar CMBs at baseline [8]. The study found that the positive rate of *APOE ε2* in CMBs was higher. Both the *APOE ε2* and *APOE ε4* alleles were associated with increased cortical CMBs [9] and white matter hyperintensity (WMH) load [10]. There is no report on the association between *APOE* genotypes and CMBs in the Chinese Han population.

A number of studies have suggested that AD7c-NTP becomes recognized as an effective biomarker for Alzheimer's disease (AD) [6]. There is an association between CMBs and AD [11]. Studies have shown that multiple CMBs or lobar and deep CMBs are associated with an increased risk of all-cause dementia [12]. Multiple lobar CMBs are involved in the rapid progression of dementia and cerebral hemorrhage [13]. The decline in cognitive abilities in patients with both vascular disease and AD is more severe than that in patients with AD [14]. It is speculated that AD7c-NTP, as an effective biomarker of AD, may be closely related to CMBs. Whether AD7c-NTP can be used as a biomarker for CMBs has not been studied. It remains unclear whether the AD7c-NTP levels interact with the *APOE* genotype. Different locations of CMBs may predict different causes and mechanisms. The lobar CMBs are mainly due to CAA, while deep or subcortical CMBs are mainly due to hypertensive microangiopathy such as lipid hyaline degeneration and fibrinoid necrosis of small vessels. Deep cerebral hemorrhage and CMBs may have the same basis of microangiopathy, and cerebral lobe hemorrhage and CMBs are due to CAA. Therefore, based on the *APOE* genotype, the correlation between urinary AD7c-NTP with CMBs was evaluated to provide effective biomarker for CMBs.

## 2. Materials and Methods

### 2.1. Study Population

**Figure 1:**
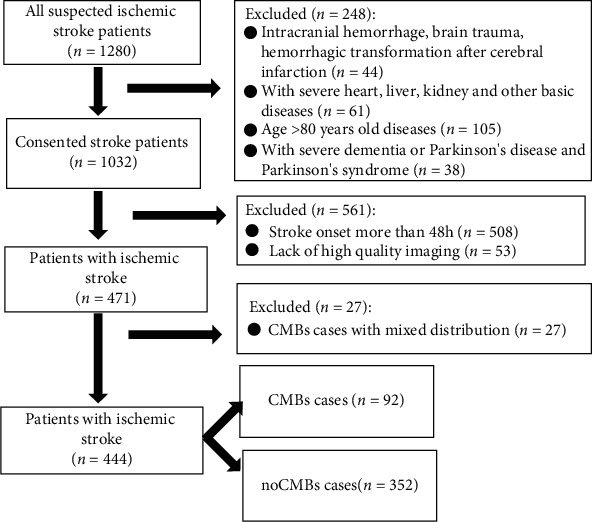
Flow chart of patients enrolled in the study.

All subjects with acute cerebral ischemic stroke enrolled in this study came from the Huangdao Branch of the Affiliated Hospital of Qingdao University and Weihai Central Hospital Affiliated to Qingdao University from August 2014 to August 2017. Inclusion criteria are as follows: (1) All cases were diagnosed in accordance with the Acute Ischemic Stroke Diagnosis and Treatment Guideline [15]. Cranial magnetic resonance imaging (MRI) confirmed a new infarction (high signal and low apparent diffusion coefficient in DWI sequence). (2) The stroke onset is less than 48 h. Exclusion criteria are as follows: (1) intracranial hemorrhage, brain trauma, hemorrhagic transformation after cerebral infarction, infection, and occupying lesions; (2) patients with severe heart, liver, kidney, pulmonary thrombocytopenia, or gastrointestinal bleeding and severe dementia or Parkinson's disease and Parkinson's syndrome; (3) age > 80 years old; (4) nervous system demyelinating diseases such as Guillain-Barre syndrome and multiple sclerosis; and (5) disturbance of consciousness. All of these were independently assessed and judged by two senior specialists. MRI (including T1W1, T2W1, DWI, and SWI) sequence tests were performed on all patients. Routine ECG, echocardiography, carotid artery ultrasound, brain MRA (or CTA), and TCD examinations were performed. The etiological classification was based on TOAST criteria. The risk factors were recorded including age, gender, smoking, alcohol consumption, Montreal Cognitive Assessment (MOCA) score, National Institutes of Health stroke scale (NIHSS) score, hypertension, systolic blood pressure, diabetes mellitus, hyperlipidemia, and antihypertensive, anticoagulant, and antiplatelet therapy use as well as thrombolysis treatment. This study has been approved by the Ethics Committee of Qingdao University. The study flow chart is showed in Figure 1.

### 2.2. Urinary Alzheimer Disease Neurofilament Protein Detection

10 ml of fasting venous blood was collected from all patients (i.e., within 72 hours of onset). Of these, 5 ml of blood was used for blood glucose, blood lipids, and blood routine and routine blood clotting tests. The middle of the morning urine 10 ml from the patients was stored at 2-8°C for the detection of AD7c-NTP. A diagnostic kit for AD7c-NTP (enzyme-linked immunosorbent assay) was used (the kit was from Shenzhen Anqun Bioengineering Co., Ltd.). The specific operation steps follow the instructions.

### 2.3. *APOE* Genotyping

Primers used for *APOE* genotyping were designed and provided by Nanjing Dongji Biotechnology Co., Ltd. In brief, DNA fragments were amplified separately, using the following primer pairs: 5′-TGTCCAAGGAGCTGCAGG-3′ and 5′-CTGCCCATCTCCTCCATCC-3′ for *APOE* rs429358r and 5′-ATGCCGATGACCTGCAGAA′ and 5′-CTGCCCATCTCCTCCATCC-3′ for *APOE* rs7412. It is given that *APOE ε*4 and *APOE ε*2 alleles are associated with a high risk of intracerebral hemorrhage and an increase of CMBs and WMH load. Different *APOE* genotypes may affect different imaging phenotypes of CMBs. Therefore, this study refers to *APOE* genotyping thoughts in previous studies. According to the different impact of *APOE* genotype on CMBs, they are divided into two categories: *ε*2 or *ε*4 allele (*ε2/ε2*, *ε2/ε3*, *ε2/ε4*, *ε3/ε4*, and *ε4/ε4*) carriers and only *ε3/ε3* allele carriers [10].

### 2.4. Neuroimaging Analysis

GE's superconducting magnetic resonance imaging system (model: GE MR Discovery 750 3.0T) was used to obtain transverse axial T1WI and T2WI, sagittal T1WI, DWI, 3D-TOF MRA, and SWI images. The scanning parameters were FLAIR-T1WI (TR/TE, 1750 ms/24 ms, TI 780 ms; FOV 24 cm × 24 cm, matrix 320 × 256), FRFSE-T2WI (TR/TE, 4300 ms/95 ms, FOV 24 cm × 24 cm, matrix 512 × 512), and DWI-EPI (TR/TE, 3000 ms/70 ms; FOV 24cm × 24 cm, matrix 160 × 160. The SWI (SWAN) sequence parameters are 3D T1-FFE, TR minimum, TE 45.0 ms, inversion angle of 15°, slice thickness of 2 mm, matrix 384 × 320, and number of excitations of 1.0. CMBs are defined as uniform low signal areas of oval or circular shape on the SWI sequence, with a diameter of 2-5 mm, no edema surrounding, and not shown on conventional sequences, except for small veins and calcifications. The diagnostic criteria for CMBs are round or elliptical on the SWI sequence, which has a low signal amplitude of 2 to 10 mm in diameter, with uniform texture, clear lesion boundaries, and no edema shadow around the lesions, and exclusion of intracranial calcification, iron deposits, cavernous hemangioma, and expanded perivascular space. According to the cerebral microbleeds anatomical rating scale, the CMBs can be divided into deep CMBs (basal ganglia, thalamus, internal capsule, external capsule and corpus callosum, and periventricular white matter), cerebral lobe CMBs (cortical and subcortical white matter), subthecal CMBs (brain stem and cerebellum), and mixed CMBs (cerebral lobe plus deep or subventral plus brain). In order to facilitate the study, the suboccipital CMBs were classified as deep CMBs in the present study. The interference of mixed CMBs was excluded in this study. All MRI images were evaluated by two experienced imaging specialists.

### 2.5. Definition of the Main Covariates

1. Hypertension: antihypertensive drugs before admission or systolic blood pressure > 140 mmHg or diastolic blood pressure ≥ 90 mmHg at admission
2. Diabetes: history of diabetes; fasting blood glucose > 7.0 mmol/l or postprandial 2 h blood glucose > 11.1 mmol/l combined glycosylated hemoglobin > 6.5%
3. Hyperlipidemia: past dyslipidemia or hospital admission abnormalities, total cholesterol > 5.72 mmol/l, TG > 1.72 mmol/l, or LDL > 3.12 mmol/l
4. Smoking: currently smoking or quitting (10 cigarettes/day for more than 5 years)
5. Drinking: daily alcohol consumption > 50 ml, alcohol abstinence or not

### 2.6. Statistical Analysis

SPSS 16.0 statistical software was used for statistical analysis. Normally distributed measurement data were expressed as the mean ± standard deviation, and count data were expressed as the percentage (%). The *t*-test was used to compare the mean of the two samples. The chi-squared test was used to compare two samples of count data. Multivariate comparisons were performed using multiple logistic analysis. *p* < 0.05 indicates that the difference was statistically significant. Logistic analysis was used to adjust the confounding factors and analyze the relationship between AD7c-NTP and imaging phenotypes of CMBs and overall CMBs. Different logistic regression models were used for different covariates. The *ε*2 and *ε*4 alleles are associated with an increased risk of lobar and deep ICH. In this study, the effect of *APOE* genotype on the susceptibility of CMBs was different, which was divided into two categories: *ε2/ε2*, *ε2/ε3*, *ε2/ε4*, *ε3/ε4*, and *ε4/ε4* and *ε3/ε3*. Through analysis of different covariates (age, gender, MOCA score, NIHSS score, hypertension, diabetes, smoking, alcohol, and antiplatelet drugs, anticoagulants, and statin). Interaction between *APOE* genotypes with AD7c-NTP was analyzed. Before the study was conducted, sample estimation and calculation of efficacy were performed. Under normal circumstances, based on previous research data, the prevalence of CMBs in patients with cerebral infarction was estimated between about 20 and 30%, the lowest value of 20% to calculate the test efficiency to take 0.8. It needs about 300-400 sample size.

## 3. Result

### 3.1. Demographic and Clinical Characteristics of the Study Subjects

This study found that the prevalence of CMBs in the cerebral infarction population was 20.7% (92/444). The majority of patients had single CMBs, of which 42 cases existed multiple CMBs. There were one hundred and nineteen CMBs cases detected including mixed distribution (cerebral lobe plus deep or under the curtain plus lobe) and 27 cases of mixed CMBs, which were excluded in order to avoid the interference to this study. The location of CMBs was more widely distributed, and 21 cases of multiple CMBs were distributed in the brain lobe. The MOCA score in the CMBs group was lower than that in the nCMBs group. Patients with CMBs from *APOE ε*4 carriers performed worse in terms of cognition (Table 1). Compared with patients with nCMBs, cerebral infarction patients with CMBs had higher *APOE ε*2 or *ε*4 allele carrier rates (*p* < 0.05).

### 3.2. Urinary AD7c-NTP Levels and CMBs

At the overall level, there was no difference in urinary AD7c-NTP levels between the nCMBs and CMBs groups (*p* > 0.05). Based on different imaging phenotypic subgroup analyses of CMBs, the level of urinary AD7c-NTP in the lobar CMBs group was significantly higher than that in the nCMBs group (*p* < 0.05). The urine AD7c-NTP levels in the *APOE ε*2 positive or *ε*4 positive patients were higher than those in the *APOE ε*3/*ε*3 genotype carriers (*p* < 0.05) in both CMBs and nCMBs patients. Urinary AD7c-NTP levels were associated with cerebral lobar CMBs (OR: 1.83, 95% C1: 1.21-3.95). In patients with cerebral lobar CMBs, there was an interaction between urine AD7c-NTP levels and *APOE* genotype (*p* = 0.01). That is to say, *APOE* genotype might be involved in the effect of urine AD7c-NTP on the risk of lobar CMBs. In patients with *APOE ε*3/*ε*3, the odds ratio for urinary AD7c-NTP levels increased by one standard deviation for cerebral lobe CMBs was 0.92 (95% CI: 0.70-1.19; *p* = 0.95). In patients with *APOE ε*2 positive or *ε*4 positive carriers, the multivariate-adjusted OR for lobar CMBs was 2.95 (95% CI: 1.38-6.27; *p* = 0.005) (Table 2).

## 4. Discussion

The incidence of CMBs was 20.7% in the present study, while the incidence fluctuates between 19.4% and 68.5% in a previous study [2]. The incidence of CMBs in patients with cerebral hemorrhage is about 38% to 66%, and cerebral infarction is about 21% to 26%, and in healthy people, it is about 5% to 6%. The incidence of CMBs in the Asian population is higher [16]. CMBs are independent risk factors for hemorrhagic transformation after acute cerebral infarction, and they are also important factors for the symptomatic hemorrhagic transformation of patients with cerebral infarction undergoing thrombolysis and anticoagulant therapy [17]. Our study observed that the prevalence of male CMBs was higher than that of females, which may be related to the older age of males in this study. For drug treatment could affect the occurrence of CMBs [16], drug use information was included in statistical analysis.

### 4.1. AD7c-NTP and the Distribution of CMBs

Urinary AD7c-NTP is associated with overall CMBs in this study. There is a relation between urine AD7c-NTP with the distribution of CMBs. That is, high levels of urinary AD7c-NTP are associated with lobar CMBs, not deep brain CMBs. Urine AD7c-NTP had high specificity and moderate sensitivity in predicting amyloid beta (A*β*) deposition among patients with cognitive impairment [18]. Lobar CMBs in Alzheimer's disease (AD) are associated with cerebral amyloid angiopathy (CAA) due to vascular A*β* deposits [19]. Deposition of amyloid appears aggravated in patients with cerebral small-vessel disease, especially in *APOE ε*4 carriers [20]. CAA is commonly seen in patients with AD, which is associated with parenchymal amyloid and may lead to WMH, microinfarcts, and MBs [21]. Furthermore, we found that urinary AD7c-NTP has certain interactions between different CMBs imaging phenotypic subgroups and *APOE* genotypes. High levels of urinary AD7c-NTP are the risk of lobar CMBs. It suggested that the *APOE* genotype and urine AD7c-NTP and other biomarkers involved in susceptibility risk control of CMBs were complex. The influence of different *APOE* allele states on the association of urinary AD7c-NTP and CMBs with different imaging phenotype risks may be mediated by different mechanisms. This mechanism may play an important role in the pathology of hypertensive cerebral vascular disease represented by deep brain CMBs.

Different imaging phenotypes of CMBs might mean different mechanisms for its occurrence. Our result also showed that the phenotype of CMBs and its susceptibility may have *APOE* allele risk dependence. The current mechanism for the development of CMBs still remains unclear, which includes many hypotheses: blood-brain barrier impairment, endothelial cell damage, deposition of beta amyloid, hypoperfusion impairment, inflammatory reactions, and genetic polymorphisms. A previous study found that urinary AD7c-NTP levels were significantly elevated in patients with mild cognitive impairment (MCI) with the *APOE ε*4 genotype [22]. In the context of *APOE* genotyping in the Han nationality, it is still unclear whether the phenotypic imaging classification of CMBs is related to urinary AD7c-NTP. It is speculated that urinary AD7c-NTP may serve as a biomarker for CMBs. Based on this hypothesis, this study first investigated the relationship between urinary AD7c-NTP and CMBs based on *APOE* genotype.

### 4.2. *APOE* Genotype Involved in the Risk Effect of Urinary AD7c-NTP for CMBs

It is generally believed that the increased risk of disease in *APOE ε*4 carriers is attributed to higher lipid levels. However, increasingly more research evidence shows that the *APOE* genotype has a direct or indirect effect on the absorption of microglial cells and microglia activation. *APOE* genotypes have different effects on mitochondrial protein expression, which may be the basis for the susceptibility of different genotypes [23]. It indicated that different *APOE* genotypes have different effects on oxidative stress and other biochemical pathological states, which have different effects on disease susceptibility [24, 25]. Therefore, studying the effect of different *APOE* genotypes on the regulation of chemokines and cytokines may help to further understand the role of ApoE-mediated cytokine regulation in the pathogenesis of CMBs. At present, the correlation between *APOE* genotype and urinary AD7c-NTP level expression is rare. In 2015, it was reported that in patients with MCI carrying *APOE ε4* genotype, serum brain-derived neurotrophic factor (BDNF) was significantly reduced, while urinary AD7c-NTP was significantly increased. Both serum BDNF and urine AD7c-NTP have higher positive predictive values and may be MCI-sensitive biomarkers [22]. The present study found that there was no difference in the levels of urinary AD7c-NTP between nCMBs and CMBs at the overall level. In subgroup analysis of different locations of CMBs, urinary AD7c-NTP levels were correlated with CMBs in the lobes. Urinary AD7c-NTP has certain interactions with *APOE* genotypes in different location CMBs. Specifically, in patients with *APOE ε*2+ or *ε*4+, high-level urinary AD7c-NTP is a risk factor for lobar CMBs.

There are several limitations: First, it is a cross-sectional study. It is unclear whether the course of acute ischemic stroke will affect the levels of urinary AD7c-NTP. Therefore, the relationship between urinary AD7c-NTP and CMBs as well as phenotypic imaging classification needs to be treated with caution. In order to minimize the impact of confounding factors, the cranial MRI was completed within 72 hours after the onset of stroke, and at the same time, blood and urine samples were collected and stored for testing. Secondly, for the differences in ethnicity, the results of this study could not be extended to other ethnic groups. Whether there are other unknown regulatory factors affecting the risk association of *APOE* genotype/CMBs remains unclear.

## 5. Conclusion

A higher level of AD7c-NTP is related to lobar CMBs. Urine AD7c-NTP may have an *APOE* genotype-dependent risk effect on CMBs. Urinary AD7c-NTP has certain interactions with *APOE* genotypes in different CMBs imaging phenotypic subgroups. In the patients with *ε*2 or *ε*4 allele carrier, the evaluation of urinary AD7c-NTP may contribute to the prediction of CMBs.

## Figures and Tables

**Table 1:** Demographics and clinical characteristics of all participants.

	No CMBs (*n* = 352)	CMBs		
		All CMBs (*n* = 92)	Lobar CMBs (*n* = 54)	Deep CMBs (*n* = 38)
Age at MRI, mean (SD)	72 (5.4)	74 (6.2)	74 (5.1)	75 (6.7)
Men, *n* (%)	168 (47.8)	48 (53.3)	30 (55.5)	18 (47.3)
MOCA (median (IQR))	27 (24-29)	27 (25-29)^a^	26 (23-29)^b^	27 (25-29)^c^
NIHSS (median (IQR))	9 (6-15)	9 (7-14)	9 (6-15)	9 (7-16)
Infarct volume (cm^3^), mean (SD)	8.5 (2.1)	8.0 (2.2)	8.2 (1.9)	7.8 (2.3)
SBP (mmHg), mean (SD)	130 (19)	135 (20)	133 (17)	139 (19)
Diabetes mellitus, *n* (%)	65 (18.5)	17 (18.5)	10 (18.5)	5 (13.2)
HDL cholesterol (mg/dl)	56 (16)	52 (18)	48 (17)	54 (19)
LDL cholesterol (mg/dl)	131 (33)	140 (36)	138 (37)	142 (35)
Current smokers, *n* (%)	24 (6.8)	7 (7.6)	5 (9.2)	3 (7.9)
Alcohol, *n* (%)	43 (12.2)	13 (14.1)	9 (16.7)	7 (18.4)
Hypertension, *n* (%)	172 (48.9)	58 (63.0)^▲^	31 (57.4)	28 (73.8)^▲▲^
Hypertension treatment, *n* (%)	140 (81.4)	45 (77.5)	23 (74.2)	22 (78.6)
Aspirin use, *n* (%)	200 (56.8)	60 (65.2)	36 (66.6)	22 (57.9)
Anticoagulant use, *n* (%)	7 (1.9)	3 (3.3)	1 (1.8)	2 (5.2)
Thrombolysis treatment	45 (12.7)	12 (13.0)	8 (14.8)	6 (15.7)
Statin use, *n* (%)	140 (39.8)	40 (43.5)	23 (42.6)	17 (44.7)
TOAST (%)				
LAA	181 (51.4)	38 (41.3)	23 (42.5)	15 (39.5)
CE	8 (3.0)	3 (4.3)	2 (4.1)	1 (5.0)
SAD	120 (45.5)	37 (53.6)	26 (53.0)	11 (55.0)
*APOE* status, *n* (%)				
*ε3/ε3*, *n* (%)	270 (76.6)	59 (64.1)	34 (62.9)	25 (65.8)
*ε2/ε2*, *ε2/ε3*, *ε2/ε4*, *ε3/ε4*, *ε4/ε4*, *n* (%)	82 (23.4)	33 (35.9)^∗^	20 (37.1)^∗∗^	13 (34.2)^∗∗∗^
AD7c-NTP (ng/dl), mean (SD)	1.05 (1.0)	1.21 (1.08)^a^	1.30 (1.11)^b^	1.08 (1.03)^c^

MOCA: ^a^*t* = 2.53, *p* = 0.005; ^b^*t* = 2.34, *p* = 0.009; ^c^*t* = 1.03, *p* = 0.15. APOE status: ^∗^*χ*^2^ = 6.0, *p* = 0.01; ^∗∗^*χ*^2^ = 4.69, *p* = 0.03; ^∗∗∗^*χ*^2^ = 2.21, *p* = 0.14. AD7c-NTP: ^a^*t* = 1.34, *p* = 0.08; ^b^*t* = 1.68, *p* = 0.04; ^c^*t* = 0.17, *p* = 0.43. Hypertension (total CMBs *vs.* nCMBs): ^▲^*χ*^2^ = 5.87, *p* = 0.016. Hypertension (lobar CMBs vs. deep CMBs): ^▲▲^*χ*^2^ = 2.56, *p* = 0.11. LAA: large artery atherosclerosis; CE: cardiogenic brain embolism; SAD: small artery disease; APOE: apolipoprotein E; NIHSS: National Institutes of Health stroke scale.

**Table 2:** Interaction between *APOE* genotype and AD7c-NTP levels in the presence of CMBs.

Model	*APOE*	*N*	Urine AD7c-NTP	*p*	Interaction
			OR (95% CI)		
Total CMBs					
1	*33*	59	0.84 (0.54-1.31)	0.44	0.49
	*22*, *23*, *24*, *34*, *44*	33	1.05 (0.53-1.32)	0.68	
2	*33*	59	0.90 (0.60-1.33)	0.60	0.45
	*22*, *23*, *24*, *34*, *44*	33	1.03 (0.94-1.12)	0.83	
Lobar CMBs					
1	*33*	34	0.83 (0.40-1.69)	0.61	0.03
	*22*, *23*, *24*, *34*, *44*	20	2.20 (1.21-3.97)	0.009	
2	*33*	34	0.92 (0.70-1.19)	0.95	0.01
	*22*, *23*, *24*, *34*, *44*	20	2.95 (1.38-6.27)	0.005	
Deep CMBs					
1	*33*	25	1.04 (0.95-1.13)	0.78	0.32
	*22*, *23*, *24*, *34*, *44*	13	1.63 (0.94-2.7)	0.08	
2	*33*	25	1.05 (0.90-1.22)	0.53	0.20
	*22*, *23*, *24*, *34*, *44*	13	1.56 (0.96-2.08)	0.07	

Odds ratio (OR) of CMBs per standard deviation change in AD7c-NTP (1SD = 1.72 for women, 1.80 for men). Model 1 adjusted for age and sex. Model 2 adjusted for age, sex, aspirin use, anticoagulant use, statin use, diabetes, APOE status (*ε*3/*ε*3 versus *ε*2/*ε*2, *ε*2/*ε*3, *ε*2/*ε*4, *ε*3/*ε*4, *ε*4/*ε*4), and systolic blood pressure.

## Data Availability

The data used to support the findings of this study are available from the corresponding author upon request.
